# A Novel Fish Pose Estimation Method Based on Semi-Supervised Temporal Context Network

**DOI:** 10.3390/biomimetics10090566

**Published:** 2025-08-25

**Authors:** Yuanchang Wang, Ming Wang, Jianrong Cao, Chen Wang, Zhen Wu, He Gao

**Affiliations:** 1 Shandong Key Laboratory of Smart Buildings and Energy Efficiency, School of Information and Electrical Engineering, Shandong Jianzhu University, Jinan 250101, China; wyc957957@163.com (Y.W.);; 2 Shandong Zhengchen Technology Co., Ltd., Jinan 250101, China

**Keywords:** fish pose estimation, underwater biomimetic robotic fish, semi-supervised learning, temporal context-aware network

## Abstract

Underwater biomimetic robotic fish are emerging as vital platforms for ocean exploration tasks such as environmental monitoring, biological observation, and seabed investigation, particularly in areas inaccessible to humans. Central to their effectiveness is high-precision fish pose estimation, which enables detailed analysis of swimming patterns and ecological behavior, while informing the design of agile, efficient bio-inspired robots. To address the widespread scarcity of high-quality motion datasets in this domain, this study presents a custom-built dual-camera experimental platform that captures multi-view sequences of carp exhibiting three representative swimming behaviors—straight swimming, backward swimming, and turning—resulting in a richly annotated dataset. To overcome key limitations in existing pose estimation methods, including heavy reliance on labeled data and inadequate modeling of temporal dependencies, a novel Semi-supervised Temporal Context-Aware Network (STC-Net) is proposed. STC-Net incorporates two innovative unsupervised loss functions—temporal continuity loss and pose plausibility loss—to leverage both annotated and unannotated video frames, and integrates a Bi-directional Convolutional Recurrent Neural Network to model spatio-temporal correlations across adjacent frames. These enhancements are architecturally compatible and computationally efficient, preserving end-to-end trainability. Experimental results on the proposed dataset demonstrate that STC-Net achieves a keypoint detection RMSE of 9.71, providing a robust and scalable solution for biological pose estimation under complex motion scenarios.

## 1. Introduction

The Earth’s surface is dominated by oceans, which constitute over 70% of the total area and contain vast reserves of natural resources. However, due to technical limitations and environmental constraints, the exploitation and utilization of marine resources remain significantly underdeveloped. As land-based resources become increasingly scarce in response to rapid population growth and rising standards of living, the strategic importance of marine resource development has never been more prominent. Particularly in China, with its extensive coastline and rich marine ecosystems, the potential for ocean-based economic and scientific advancement is substantial. Yet, the dynamic and complex nature of underwater environments imposes considerable challenges for human intervention and exploration.

To overcome these limitations, underwater robots have emerged as a viable solution for performing tasks in hazardous or inaccessible underwater settings. These systems are widely applied in fields such as marine oil and gas extraction, underwater archaeology, environmental monitoring, and marine scientific research. However, most conventional underwater robots rely on electric propeller-based propulsion systems, which are often structurally complex, energetically inefficient, and limited in maneuverability. In contrast, fish have evolved over millions of years to exhibit exceptional hydrodynamic performance and adaptability, making them an ideal model for biomimetic underwater robotic design. By emulating fish locomotion strategies, engineers aim to develop next-generation underwater robots that offer superior efficiency, agility, and environmental adaptability.

A key technological enabler for such biomimetic systems is pose estimation, a computer vision technique that identifies the spatial configuration of objects or organisms in images or video frames. Although significant progress has been made in human pose estimation driven by deep learning advancements, animal pose estimation—particularly for aquatic species—remains comparatively underexplored. This gap stems from several intrinsic challenges, including the anatomical diversity across species, the high variability and speed of animal motion, the labor-intensive nature of data annotation, and environmental complexities such as occlusions and dynamic lighting in natural habitats. Moreover, existing datasets and algorithmic toolchains tailored for animal pose estimation are still in early stages of development.

To address these challenges, a series of foundational studies have investigated fish motion mechanisms through image processing, 3D reconstruction, and hydrodynamic analysis. Early works, such as Jing et al. [1], examined crucian carp motion dynamics, identifying distinct behavioral stages via hydrodynamic modeling. Li et al. [2] highlighted the importance of dorsal fin oscillation in propulsion for the Nile electric eel, while Yan et al. [3] and Wu et al. [4] developed multi-angle video systems to analyze swimming trajectories and kinematics of cyprinids and koi carp. Lai et al. [5] and Stern et al. [6] advanced behavioral detection systems and landmark recognition methods based on image processing and machine learning, respectively.

Subsequent studies introduced 3D pose reconstruction techniques based on stereo vision [7,8,9,10], and deep learning approaches for robust fish tracking and classification [11,12,13,14,15]. Lin et al. [16] developed the first fish pose dataset with 1000 annotated images and proposed a two-stage estimation framework, achieving over 90% detection accuracy. Further innovations have emerged in pose recovery under occlusion [17], monocular 3D reconstruction [18], and spatiotemporal modeling using LSTM networks [19]. Lightweight deep learning models, such as MFLD-net [20], and graph-based behavior detection frameworks [14], have improved computational efficiency and detection robustness in aquaculture scenarios.

Meanwhile, several researchers have explored skeleton-based 3D tracking [21], stereoscopic keypoint detection [22], and motion parameter estimation [23], enabling more precise modeling of fish dynamics. Pose estimation methods leveraging DeepLabCut [24] and other deep learning architectures have shown promise in extracting motion parameters like velocity, acceleration, and angular displacement with high accuracy. These studies collectively reflect a growing trend towards integrating visual perception, behavior modeling, and biomechanical analysis for intelligent underwater systems.

Beyond fish-focused studies, related advances in representation learning are also relevant to this work. For instance, the study by Zhang et al. [25] investigated strategies for balancing multi-task learning objectives, and its insights on task interaction inspired our consideration of how joint feature optimization can improve pose estimation performance. In addition, the work of Li et al. [26] addressed representation learning under incomplete multi-view scenarios, highlighting the importance of leveraging structural consistency across multiple feature views, which is conceptually related to the spatiotemporal consistency emphasized in our method.

Despite these advancements, limitations still exist in data quality, generalization under occlusion, and temporal modeling of continuous fish motion. Moreover, there remains a lack of unified frameworks that can bridge fine-grained pose estimation with real-time feedback control for robotic applications. In response to these gaps, this paper proposes a high-precision fish motion capture and analysis framework combining a custom-built dual-camera acquisition system with a deep learning-based semi-supervised pose estimation network. The platform captures multi-view sequences of carp performing natural swimming behaviors and annotates 21 anatomical keypoints for high-fidelity motion reconstruction. Building on this dataset, the proposed Semi-supervised Temporal Context-Aware Network (STC-Net) fuses spatial and temporal information across frames and integrates novel unsupervised loss functions to enhance performance under limited supervision.

This study contributes not only a valuable dataset and technical framework for fish pose estimation but also provides a scalable solution for real-time pose tracking in underwater biomimetic robotic control systems. The proposed approach holds significant potential for applications in marine resource development, aquaculture monitoring, and autonomous underwater navigation.

The main contributions of this paper are as follows:

- To address the critical limitation posed by the scarcity of high-quality fish motion posture datasets, we have developed a custom-designed fish motion visualization experimental platform. The system consists of a transparent water tank (dimensions: 120 cm × 60 cm × 60 cm), a high-performance computing workstation, dual synchronized cameras, and two auxiliary lighting sources. By synchronously capturing multi-view motion sequences of fish from orthogonal perspectives, the platform enables precise annotation and facilitates the construction of a multi-view, fine-grained fish posture dataset, thereby providing foundational data support for downstream pose estimation tasks.
- To enhance the robustness of pose estimation under conditions of occlusion and motion ambiguity, we introduce a novel architectural modification to the fully supervised baseline model by incorporating a Bidirectional Convolutional Recurrent Neural Network (Bi-ConvRNN) into the head of the network. This module fuses spatial-temporal features from adjacent frames—specifically, two preceding and two succeeding frames relative to the labeled target frame—allowing the model to capture motion continuity and spatial correlation across time. This temporal context integration significantly improves prediction accuracy in scenarios where the target fish body parts are partially or fully occluded.
- We propose an enhanced loss function framework by introducing two unsupervised loss terms: the temporal continuity loss, which enforces consistency in predicted keypoint trajectories across consecutive frames, and the pose plausibility loss, which constrains predicted poses to adhere to biologically valid configurations. During training, both labeled and unlabeled frames are fed into the network. The unsupervised loss terms enable effective utilization of unlabeled data, thereby improving generalization performance and reducing the reliance on large-scale manual annotations. This semi-supervised learning strategy ensures greater scalability and applicability of the model in real-world underwater environments.

## 2. Fish Pose Estimation with a Semi-Supervised Temporal Context-Aware Network

### 2.1. Fully Supervised Paradigm and Its Limitations

#### 2.1.1. Standard Fully Supervised Fish Pose Estimation Model

Currently, widely used frameworks for animal pose estimation, such as DeepLabCut [27], SLEAP [28], and DeepPoseKit [29], predominantly adopt a fully supervised learning paradigm based on heatmap regression to infer animal poses on a frame-by-frame basis. Despite differences in network architecture and implementation, these methods share a common methodological foundation: they depend on densely annotated datasets and learn to localize keypoints by minimizing the discrepancy between predicted and ground-truth heatmaps. In terms of representative approaches, DeepLabCut builds upon a ResNet backbone with transfer learning, which provides powerful feature extraction but is more vulnerable to occlusion and background noise. SLEAP employs a UNet-style encoder–decoder architecture that is well-suited for multi-animal tracking, though it sometimes exhibits higher localization variance when individuals are in close proximity. DeepPoseKit is another relevant supervised framework, but its performance in complex 3D environments and multi-animal interaction scenarios is generally weaker compared to DeepLabCut and SLEAP.

**Figure 1:**
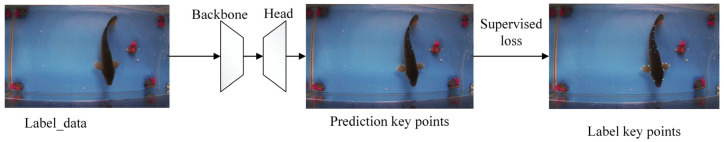
Standard supervision network diagram.

This conventional supervised pipeline typically comprises two main components. The first is the backbone network, which extracts high-level spatio-temporal features from each input frame. The second is the head module, which leverages these features to generate heatmaps corresponding to anatomical landmarks, as illustrated in Figure 1. While effective, this paradigm requires extensive manual labeling, thereby limiting scalability and adaptability to new species or behaviors. These limitations motivate the exploration of more efficient and generalizable alternatives, including semi-supervised and self-supervised approaches.

The Backbone module plays a pivotal role in the pose estimation framework by extracting hierarchical and semantically rich feature representations from input images or video frames. These extracted features serve as the foundation for the downstream estimation process. Given the remarkable capability of deep convolutional neural networks (CNNs) in visual representation learning, architectures such as ResNet and EfficientNet have emerged as widely adopted backbone choices in recent animal pose estimation studies. Their depth and architectural efficiency enable the model to capture intricate spatial relationships critical for accurate keypoint localization.

Complementing the Backbone, the Head module is tasked with transforming the high-dimensional feature maps into pose-specific outputs. Concretely, the Head generates a series of heatmaps, where each heatmap corresponds to a specific anatomical landmark or body part. These heatmaps are formulated as two-dimensional probability distributions, with higher intensity values indicating a higher likelihood of the presence of the corresponding body part at a given location in the image.

In the context of fish pose estimation, this study systematically evaluates the effectiveness of various ResNet variants as the Backbone architecture. To assess and compare the performance of these networks, we employ Root Mean Square Error (RMSE) as the evaluation metric, quantifying the average deviation between predicted and ground-truth keypoint locations. The comparative results are detailed in Table 1**,** providing insight into the relative accuracy of different network configurations under the same experimental conditions.

As shown in Table 1, we conduct a comparative performance evaluation of three widely used variants of the ResNet architecture—ResNet-34, ResNet-50, and ResNet-101—within the context of the fish pose estimation task. The experimental results indicate that ResNet-50 achieves a favorable trade-off between computational efficiency and prediction accuracy. Specifically, ResNet-50 attains an RMSE of 14.50, which is significantly lower than that of the shallower ResNet-34, demonstrating improved representation capability. Although ResNet-101 achieves the lowest RMSE of 14.03, the marginal improvement in accuracy comes at the cost of a notable increase in computational complexity. Therefore, considering accuracy, computational efficiency, and deployment scalability, ResNet-50 is selected as the baseline Backbone architecture for subsequent experiments in this study.

**Figure 2:**
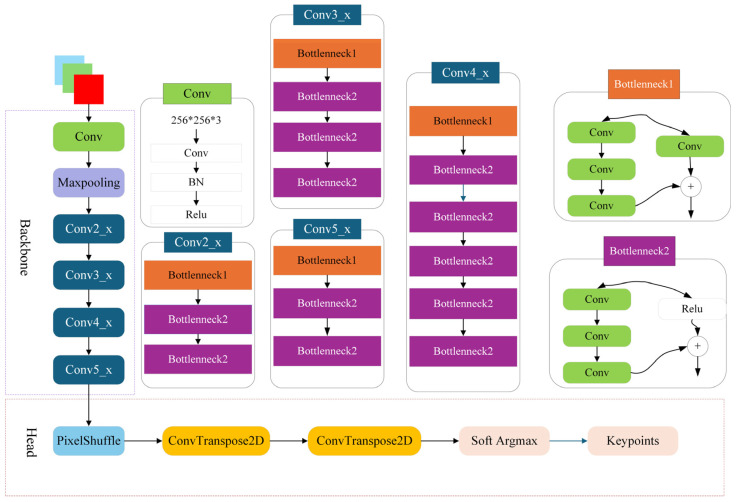
Fully Supervised Network Structure Diagram.

The architecture of the proposed fully supervised fish pose estimation network is illustrated in Figure 2, which comprises two key components: the Backbone and the Head. The Backbone adopts the ResNet-50 architecture to extract multi-scale, high-level semantic features from input images. Each input is a 256 × 256 × 3 RGB frame, which first passes through a 7 × 7 convolutional layer followed by a 3 × 3 max pooling layer. This is succeeded by four residual blocks, consisting of 3, 4, 6, and 3 Bottleneck units, respectively. This hierarchical processing results in a compact yet semantically rich feature map of size 8 × 8 × 2048.

The Head module is designed to progressively upsample the low-resolution feature map into a high-resolution output heatmap. This is achieved through a combination of PixelShuffle operations and ConvTranspose2D layers, enabling precise spatial recovery of keypoint information. Finally, to enhance localization accuracy, the Soft Argmax function is applied to the generated heatmaps to extract sub-pixel keypoint coordinates, thereby enabling high-precision pose estimation.

#### 2.1.2. Issues in Fully Supervised Animal Pose Estimation

Although fully supervised animal pose estimation frameworks leverage extensive labeled training data and standard post-processing techniques, they are not immune to prediction errors. In practice, pose estimation outputs may still exhibit anomalies or inaccuracies, particularly in complex motion sequences or under challenging visual conditions. In this study, we manually annotated 420 frames from a custom-collected carp video dataset and trained a pose estimation model using the SLEAP framework. The model successfully converged during training, indicating effective learning of spatial representations for fish body keypoints.

**Figure 3:**
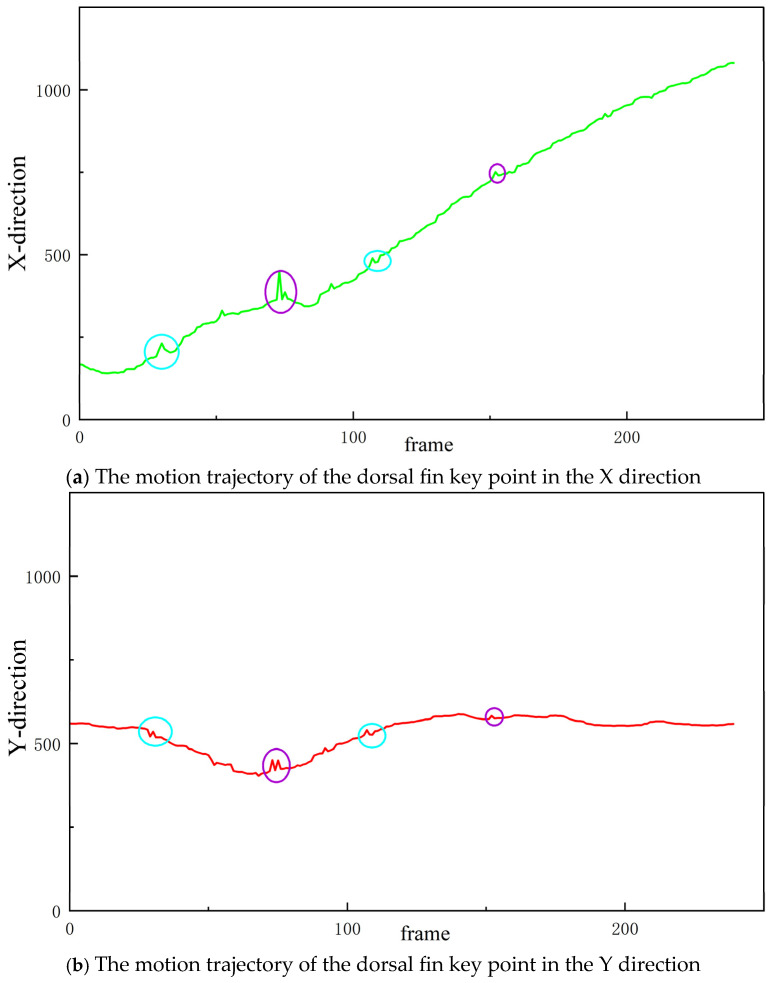

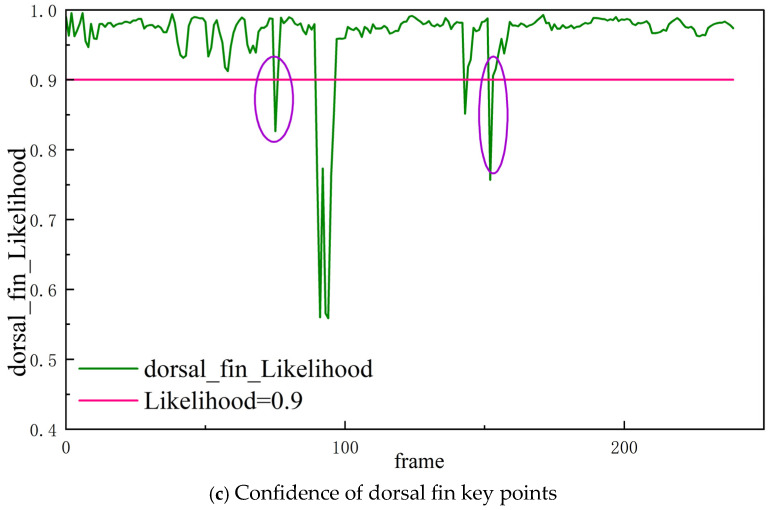
SLEAP algorithm’s predicted trajectory for the dorsal fin keypoints.

Figure 3 illustrates a sequence of predicted positions for the dorsal fin keypoint across continuous video frames, along with the corresponding confidence scores provided by the SLEAP network. Analysis of these confidence trajectories reveals two distinct categories of anomalous predictions. The first category consists of low-confidence outliers, highlighted with purple circles. These points exhibit abrupt deviations from the overall trajectory and are accompanied by confidence scores below a pre-defined threshold (e.g., <0.9). Such outliers are relatively straightforward to detect and eliminate via simple confidence-based filtering, as the model itself assigns low reliability to these estimates. The second category involves high-confidence but semantically incorrect predictions, highlighted with blue circles. These points represent a more challenging class of errors: although the predicted positions cause unnatural or biologically implausible discontinuities in the keypoint trajectory, the model assigns high confidence scores, falsely indicating prediction reliability. Such errors typically arise due to mislocalization in visually ambiguous frames or occlusion, where the model confuses spatial context and outputs keypoints that diverge significantly from the true anatomical path.

This analysis highlights a key limitation of confidence-based filtering mechanisms: while effective against low-confidence noise, they fail to detect high-confidence mispredictions. Addressing such errors requires more robust spatiotemporal consistency checks or post hoc correction mechanisms, which motivates the subsequent design innovations in this work.

### 2.2. Semi-Supervised Temporal Context-Aware Network

#### 2.2.1. Temporal Context-Aware Network

Accurate keypoint localization becomes particularly challenging when the target anatomical point is occluded by other body parts or lies in visually ambiguous regions. Additionally, the presence of morphologically similar body structures can lead to misidentification, further complicating prediction accuracy. In such cases, relying solely on spatial information from a single frame is often insufficient. However, incorporating temporal context, i.e., visual cues from adjacent frames, offers a promising avenue for improving robustness. For instance, when a keypoint is occluded in the current frame, its position can often be inferred from its trajectory in preceding and subsequent frames, effectively allowing the model to interpolate or “fill in” missing information.

Despite the inherent availability of such temporal patterns in video data, most existing pose estimation models operate under a frame-by-frame paradigm, leveraging only spatial information within individual frames and disregarding the rich temporal continuity present in labeled sequences. To address this limitation, we propose a novel architecture termed the Temporal Context Network (TCN), specifically designed to harness temporal cues for improved keypoint estimation.

**Figure 4:**
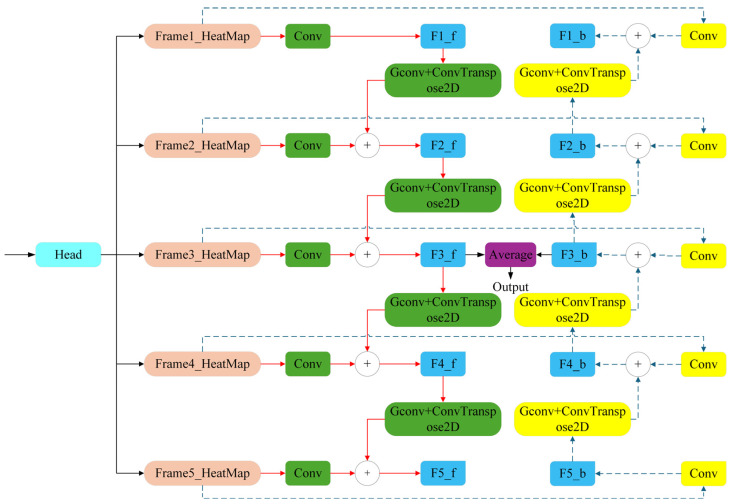
Bidirectional convolutional recurrent neural network structure.

The architecture of the proposed temporal context-aware network is illustrated in Figure 4 The network takes as input a five-frame sequence and predicts the keypoint heatmap for the central (third) frame. Similar to standard pose estimation pipelines, each frame is first passed through a shared Backbone network to extract spatial feature representations. However, unlike conventional models that treat each frame independently, the temporal context-aware model aggregates multi-frame features via a Bidirectional Convolutional Recurrent Neural Network (Bi-ConvRNN) module, which models both forward and backward temporal dependencies.

The Bi-ConvRNN effectively integrates the strengths of Convolutional Neural Networks (CNNs) for spatial feature extraction and Recurrent Neural Networks (RNNs) for temporal sequence modeling. Compared to naive temporal stacking or 3D convolution methods, Bi-ConvRNN offers a lightweight and efficient alternative by avoiding redundant computation across frames. While the Backbone extracts spatial features from individual frames, the Bi-ConvRNN module focuses on learning the temporal dynamics across the sequence, enabling more accurate and temporally consistent pose predictions.

Figure 4 provides a detailed schematic of the proposed Bidirectional Convolutional Recurrent Neural Network (Bi-ConvRNN) module, which serves as the core component of the temporal context-aware pose estimation architecture. The network operates on a sequence of five consecutive heatmaps, corresponding to the same keypoint across five adjacent video frames. Its objective is to leverage temporal dependencies from both the preceding and subsequent frames to improve the accuracy of keypoint localization in the central (third) frame.

The pipeline begins with a preliminary upsampling of the input heatmaps via a pixel rearrangement (PixelShuffle) operation, ensuring sufficient spatial resolution for downstream temporal modeling. When necessary, additional upsampling layers are applied to further enhance spatial fidelity. The processed heatmaps are then split into two branches: (i) A forward temporal branch (denoted by red solid arrows) propagates information from the first frame to the last. (ii) A reverse temporal branch (denoted by blue dashed arrows) flows in the opposite direction, from the fifth frame to the first.

In each branch, the heatmap of the initial frame is transformed into feature representations using convolutional layers. Subsequent frames are then processed recursively, with each step combining spatial features from the current frame and temporal information from the previous step. This recurrent process enables the model to accumulate temporal knowledge across the sequence. After both temporal branches have traversed their respective directions, their outputs are fused at the central frame via pixel-wise averaging, producing the final, high-precision heatmap. Keypoint coordinates are then extracted by applying spatial normalization followed by maximum response localization.

Despite the availability of large volumes of unlabeled video data, most existing animal pose estimation methods fail to utilize such data effectively during training. These models typically rely on supervised learning paradigms, focusing on frame-wise localization using only labeled samples, and incorporating video data solely during inference. Consequently, the potential of rich spatiotemporal patterns present in unlabeled sequences remains underexploited.

#### 2.2.2. Loss Function Improvement

To overcome this limitation, we propose a semi-supervised training strategy that enhances the fully supervised pose estimation model by integrating two unsupervised loss functions: Temporal Continuity Loss-penalizes abrupt and implausible variations in keypoint trajectories across consecutive frames; Pose Rationality Loss-constrains the predicted pose to remain within anatomically feasible configurations.

During training, unlabeled continuous video clips are fed into the network alongside labeled data. When the predicted keypoint positions violate the defined temporal or anatomical constraints, the network receives penalty signals that are backpropagated to update model weights. Importantly, these unsupervised loss terms are only active during training and have no impact on inference speed, thus maintaining the runtime efficiency of the original fully supervised architecture.

**Figure 5:**
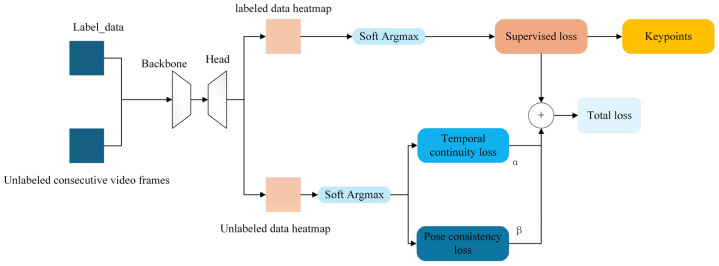
Semi-supervised network fish posture estimation model.

The resulting semi-supervised architecture, which incorporates both labeled and unlabeled data streams through spatiotemporal regularization, is depicted in Figure 5. This approach significantly enhances the model’s robustness and accuracy, particularly in occluded or visually ambiguous scenarios, without increasing the inference-time computational cost.

The network structure diagram shows a semi-supervised learning framework for pose estimation tasks. The network receives two types of input: labeled data with true data labels, and unlabeled continuous video frames. Both labeled and unlabeled data are processed by the Backbone and Head modules to generate heat maps. For labeled data, the heat map is converted to keypoints coordinates by the Soft Argmax module, which is used to calculate the fully supervised loss (RMSE), which measures the difference between the predicted heat map and the true heat map. The heat map of unlabeled data is also processed by the Soft Argmax module, and its output is used to calculate the temporal continuity loss and the pose reasonableness loss, which respectively ensure the temporal consistency of pose estimation between consecutive frames and the pose estimation is consistent with the animal’s anatomical structure and movement pattern. Finally, the fully supervised loss, temporal continuity loss, and pose reasonableness loss are weighted and summed to form a total loss. The network parameters are optimized by backpropagation to improve the performance of the model in the pose estimation task. The formula is shown in the following. The two unsupervised loss functions will be introduced below.

(1) $$L_{total}=L_{RMSE}+\alpha L_{T}+\beta L_{P}$$

where α and β default to 1. $L_{RMSE}$ represents fully supervised loss. $L_{T}$ represents time continuity loss, $L_{P}$ represents attitude rationality loss.

First, the first unsupervised loss is the temporal continuity loss, whose theoretical basis is derived from the spatiotemporal continuity characteristics of object movement, that is, the movement of an object should be continuous, that is, the object will not jump too much in a continuous video frame. In this paper, pixel distance is used to measure the jump amplitude of key points, and the Euclidean distance of each key point in consecutive video frames is calculated. Considering the differences in motion characteristics of different key points, different thresholds are set for different key points to ensure that the model can ignore small motion noise within a reasonable range while being sensitive to abnormal motion mutations, thereby reducing computational redundancy and ensuring the rationality of motion estimation. The calculation process of temporal continuity loss $L_{t}(\varepsilon )$ is shown in Equation (2).

(2) $$L_{t}(\varepsilon )=\max(0,{\left\|y_{k}^{t}-y_{k}^{t-1}\right\|}_{2}-\varepsilon )$$

where $y_{k}^{t}$ is the predicted position of the *k* keypoint in the t frame, and ε is the threshold set.

In the task of posture estimation, the key points predicted by the network may have the following problems: the positions of some key points may be beyond the biologically feasible range, such as the reverse bending of the arm. Although mathematically the key points can move freely in the 2K-dimensional space, the real movement is usually restricted to fewer degrees of freedom. If only time loss (Euclidean distance) is used for supervision, the model may learn unreasonable postures. Researchers introduced the concept of low-dimensional motion prior, modeling reasonable posture distribution through statistical methods or deep learning technology, and imposed constraints during network training to ensure that the prediction results conform to the laws of biological motion. Bialek et al. [29] quantitatively analyzed how to face the challenges of high-dimensional data, how to simplify such data by finding low-dimensional structures, and how to understand and define the dimensions of behavior.

The core idea of Posture plausibility loss is that although the mathematical representation of key points is in 2K-dimensional space, their actual movement is often restricted to a lower-dimensional subspace (for example, human gait may be mainly controlled by 3–5 latent variables). By performing principal component analysis (PCA) on a large amount of annotated data, a low-dimensional motion subspace can be learned, and a loss function can be used to constrain the posture predicted by the network to this subspace. Assuming that a posture consists of K key points, each key point is a 2D coordinate, then a high-dimensional posture vector is obtained, as shown in Equation (3).

(3) $$Y_{P-PCA}=\left(\begin{array}{c} y_{1}^{T}\\ y_{2}^{T}\\ \vdots \\ y_{N}^{T} \end{array}\right)$$

where *N* represents the number of key points, $y_{i}$ represents the 2D coordinate concatenation vector of all key points in the frame.

If the pose of some frames is missing (for example, some body parts are occluded), the row will be removed. Since the complete pose vector is used instead of the coordinates of a single key point, the PCA training samples need to satisfy $N_{train}\geq 2K$, that is, the number of training samples must be at least equal to the total number of key point coordinates, otherwise PCA cannot be effectively modeled.

For the selection of principal components, there is no longer a limit of retaining three principal components. Instead, the number of principal components R that can explain 99% of the variance is retained as needed. These principal components constitute a new matrix P, as shown in Equation (4). Each principal component represents a reasonable whole-body movement pattern.

(4) $$P=\left[\begin{array}{cccc} P_{1} & P_{2} & \ldots & P_{R} \end{array}\right]$$

where R means that the high-dimensional posture vector $Y_{P-PCA}$ is obtained by dimensionality reduction to obtain the principal component matrix P, and each principal component $P_{i}$ represents a typical whole-body movement pattern.

During the training process, the network will generate a complete posture prediction $\overset{\wedge }{y_{t}^{k}}$, which is the posture prediction for the t-th frame and the k-th body part. It is a 2K-dimensional vector. This 2K-dimensional prediction is projected onto a low-dimensional hyperplane, and then restored to the 2K-dimensional space to obtain PCA and the reconstructed posture after projection. The specific calculation formula is shown as follows.

(5) $$\overset{-}{y_{t}^{k}}=(\overset{\wedge }{y_{t}^{k}}-\mu )PP^{T}+\mu$$

where μ is the mean vector of the training data, which represents the average posture of all training data.

If the posture $\overset{\wedge }{y_{t}^{k}}$ predicted by the network is consistent with the true posture, then the reconstructed posture $\overset{\wedge }{y_{t}^{k}}$ after PCA projection should be very close to the predicted value $\overset{\wedge }{y_{t}^{k}}$. The core idea of posture rationality loss is that the posture $\overset{\wedge }{y_{t}^{k}}$ predicted by the network should be kept in a low-dimensional space that conforms to the laws of biological motion. If the predicted posture $\overset{\wedge }{y_{t}^{k}}$ is already on a reasonable posture distribution hyperplane, that is, $\overset{\wedge }{y_{t}^{k}}$ = $\overset{-}{y_{t}^{k}}$, the reconstruction is consistent with the original prediction and no additional error will be generated; if the posture predicted by the network deviates from this space, a loss will occur. The loss function is defined as the pixel error between the predicted posture $\overset{\wedge }{y_{t}^{k}}$ and the reconstructed posture $\overset{-}{y_{t}^{k}}$. The calculation formula of the loss function is shown in Formula (6).

(6) $$L_{t,k}^{P-P}(\varepsilon )=\max(0,{\left\|\overset{\wedge }{y_{t}^{k}}-\overset{-}{y_{t}^{k}}\right\|}_{2}-\varepsilon )$$

where ε is selected by performing PCA reconstruction on the annotated pose vector, calculating the pixel error of each 2D key point, and taking the maximum value.

## 3. Experimental Results and Analysis

**Figure 6:**
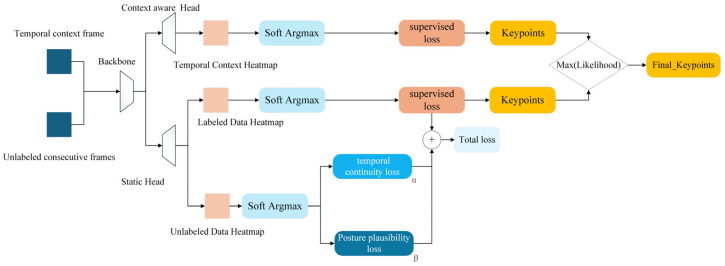
Overall structure diagram.

Since the temporal context-aware network and the unsupervised loss act independently (one requires modifying the network architecture and the other requires modifying the loss function), the two can be seamlessly combined to form a semi-supervised temporal context-aware network. For the labeled frames, a continuous image sequence of 5 frames is used to predict the pose of the intermediate frame, which is then compared with the true value; at the same time, the unlabeled video data also uses a continuous image sequence of 5 frames to generate predictions of the intermediate frames, but there is no corresponding true value label, so the unsupervised loss is applied to these predictions. The overall structural block diagram is shown in Figure 6.

### 3.1. Datasets

**Figure 7:**
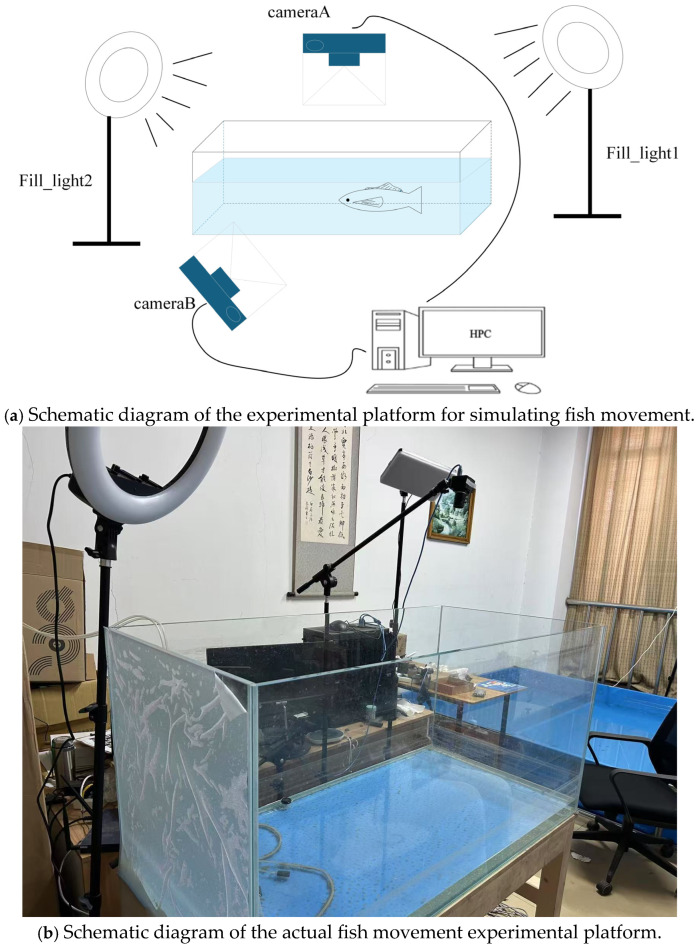
Fish movement posture experimental platform.

**Figure 8:**
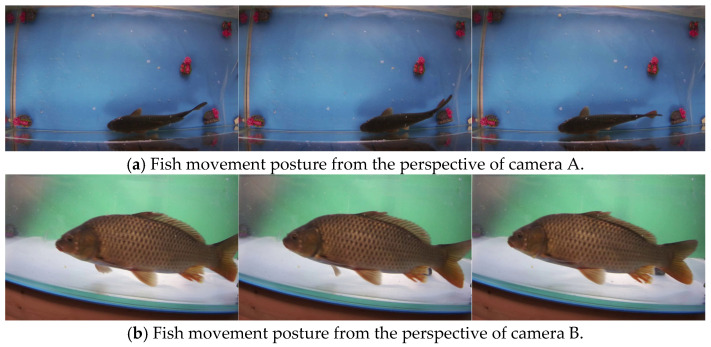
Fish movement posture dataset collected by the experimental platform.

Most of the existing fish data sets focus on fish classification, while the original intention of this study is to systematically summarize the movement rules of fish through in-depth analysis of fish movement postures, so as to guide the posture control of bionic robot fish in underwater environments. Therefore, most existing fish data sets are not suitable for the research objectives of this paper. Therefore, in order to study fish movement, a fish posture and motion estimation platform was built. The platform mainly includes the collection, processing and visualization of fish movement data. The experimental platform includes: a carp (350 mm long), a fish tank (120 cm × 60 cm × 60 cm), a high-performance computer, two synchronous cameras, and two fill lights. The experimental platform is shown in Figure 7. The movement of the fish in the pool is captured by two cameras at the same time and stored on the computer hard disk. The fill light can provide uniform light for the entire recording process. With the help of this experimental platform, a total of 16 videos were shot, each of which is no less than 2 min. Part of the data set is shown in Figure 8.

### 3.2. Experimental Environment

The proposed semi-supervised temporal context-aware network for fish pose estimation represents a significant advancement over standard fully supervised models. This method integrates two core enhancements: (1) the incorporation of a temporal context modeling module, and (2) the introduction of unsupervised loss functions for learning from unlabeled video sequences. By seamlessly combining temporal modeling with a semi-supervised learning paradigm, the framework is capable of capturing both spatial and temporal dependencies while leveraging large volumes of unlabeled data to improve prediction accuracy and robustness. This architecture effectively addresses several limitations of conventional approaches, including the reliance on densely labeled datasets, the lack of temporal reasoning, and vulnerability to occlusions or anatomical ambiguities. Through the joint optimization of supervised and unsupervised objectives, the model learns temporally coherent and anatomically plausible keypoint trajectories without sacrificing inference efficiency.

The implementation details and hardware configuration of the experimental platform used for model training and evaluation are summarized in Table 2.

### 3.3. Network Training

The fish pose estimation experiments conducted in this study are based on a self-constructed carp dataset, specifically curated to support the development and evaluation of the proposed semi-supervised temporal context-aware network. The dataset consists of 400 manually annotated static images and 20 unlabeled video sequences, each with a duration of 10 s, capturing various postures and motion patterns of carp in natural aquatic environments. This dataset provides both spatially labeled frames for supervised learning and temporally continuous sequences for unsupervised learning, enabling comprehensive evaluation under the semi-supervised learning paradigm.

During training, each input image or video frame is resized to 384 × 384 pixels. The Backbone of the pose estimation network adopts the ResNet-50 architecture, pre-trained on the ImageNet dataset to accelerate convergence and improve feature representation quality. The network is optimized using the Adam optimizer [30], which offers adaptive learning rates for different parameters and generally improves training stability.

A dynamic learning rate adjustment strategy is employed: the initial learning rate is set to 0.001, and is subsequently reduced by a factor of 0.5 at epoch 150, epoch 200, and epoch 250, respectively. To ensure stable convergence and prevent early disruption of the pretrained feature extractor, the weights of the ResNet-50 Backbone are frozen during the first 20 epochs. The batch size is set to 6, and the total number of training epochs is 300.

This carefully designed training protocol balances learning stability and flexibility, allowing the model to effectively learn both spatial localization from labeled data and temporal-spatial regularities from unlabeled sequences.

### 3.4. Results Analysis

The experimental design mainly includes ablation experiments and comparative experiments. The ablation experiment aims to evaluate the impact of different improvements on the model training results, while the comparative experiment is used to verify the effectiveness of the algorithm in this paper and conduct an in-depth analysis of the training results before and after the improvement.

(1) Ablation experiment

In order to verify the effect of the added module on improving network performance, this section designs an ablation experiment. First, the standard fully supervised network (Base network) is trained as the baseline model. Then, improvements are made on the basis of the Base network: first, unsupervised loss is added to build a semi-supervised network (Semi-Supervised network); second, the time context perception mechanism is introduced to form a time-aware contextual network (Time-aware Contextual Network). Finally, the unsupervised loss and the time context perception mechanism are simultaneously integrated into the Base network to build a semi-supervised time-aware contextual network (Semi-Supervised Time-aware Contextual Network) to further verify the role of the hybrid module. The results of the ablation experiment are shown in Table 3.

As summarized in the preceding table, the proposed semi-supervised temporal context-aware network achieves the best performance in the fish pose estimation task, yielding a Root Mean Square Error (RMSE) of 9.71 pixels. This represents a significant reduction of 4.41 pixels compared to the baseline fully supervised network, demonstrating a substantial enhancement in prediction accuracy. The incorporation of unsupervised loss functions alongside the use of unlabeled video frames during training enables the model to learn richer spatiotemporal patterns, thereby improving its generalization capability and reducing estimation errors.

The temporal context-aware network, which inputs a triplet of consecutive frames (the target frame along with its immediate predecessor and successor), benefits from leveraging adjacent temporal information to inform keypoint predictions. However, when compared to the semi-supervised model, its error reduction is more modest, suggesting that temporal information alone is insufficient to fully resolve ambiguities or occlusions inherent in fish posture estimation. By contrast, the semi-supervised temporal context-aware network synergistically combines the strengths of semi-supervised learning and temporal modeling. This hybrid approach not only exploits unlabeled data to enhance model robustness but also incorporates temporal continuity to stabilize predictions across frames. Consequently, the proposed framework significantly improves pose estimation accuracy and attains the best overall performance.

**Figure 9:**
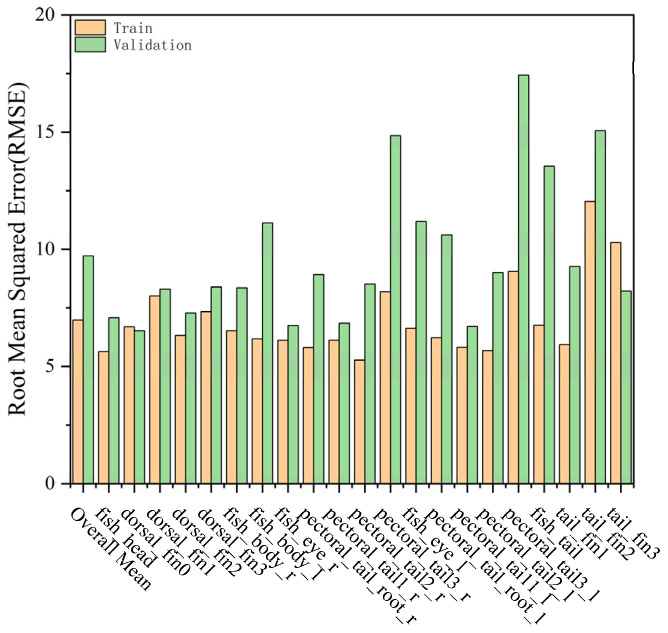
Histogram of the Root Mean Squared Error (RMSE) for fish keypoints.

These experimental findings underscore the effectiveness of jointly leveraging semi-supervised learning and temporal context information as a strategic solution for advancing fish pose estimation accuracy. Figure 9 illustrates the RMSE values for individual fish body parts evaluated on both the training set and the validation set. The relatively low RMSE observed for the overall mean across both datasets indicates the model’s strong generalization and reliable predictive capability for diverse anatomical landmarks.

**Figure 10:**
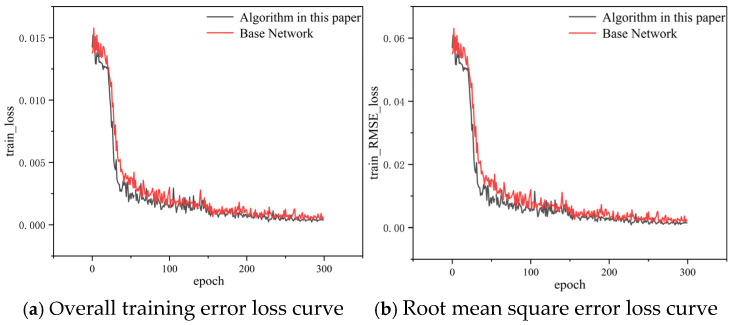
Loss function comparison curve.

Figure 10 shows the loss as a function of Epoch for two models: the semi-supervised temporal context-aware network (black curve) and the standard network (red curve) on the training set. Both curves exhibit a downward trend, indicating that the models successfully reduced errors and improved their fitting ability during training. In the initial training phase (approximately 0~50 epochs), both models show a sharp decrease in loss, suggesting that the models quickly learned the key features. However, the loss curve of the temporal context-aware network drops more rapidly, indicating that the network learned more effective features. After 50 epochs, the training loss becomes stable, suggesting that the models gradually converged. Yet, the loss of the semi-supervised temporal context-aware network remains lower than that of the standard network, indicating that its training error is smaller and its fitting ability is stronger.

**Figure 11:**
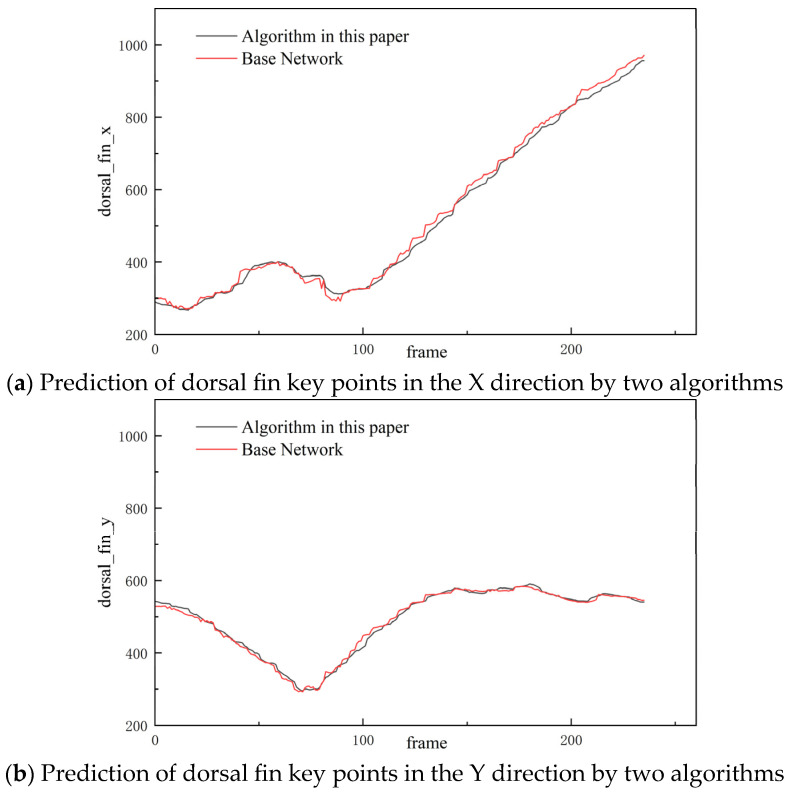
Prediction of the same keypoint trajectory by two models.

Figure 11 shows the prediction of the key point trajectory of the carp dorsal fin by the base model and the semi-supervised time context-aware network. The red curve represents the prediction of the base model, and the black curve represents the prediction trajectory of the semi-supervised time context-aware network. The first sub-figure shows the prediction of the dorsal fin key point in the X direction by the two models, and the second sub-figure shows the prediction of the dorsal fin key point in the Y direction by the two models. As can be seen from the figure, the predicted trajectory of the base model has more burrs and jumps, while the key point trajectory predicted by the semi-supervised time context network is smoother, the outliers are significantly reduced, and the overall performance is more stable.

(2) Comparison experiments

To verify the effectiveness of the algorithm proposed in this paper, we selected the proposed algorithm and compared it with two representative fully supervised algorithms, DeepLabCut and SLEAP (both trained with default parameters as per their official implementations), on the fish pose dataset built in this paper. The models were trained and their performance was analyzed. The experimental results are shown in Table 4.

As evidenced by the experimental data, the proposed algorithm significantly outperforms both DeepLabCut and SLEAP in terms of Root Mean Squared Error (RMSE). Specifically, DeepLabCut and SLEAP achieve RMSE values of 15.21 pixels and 14.84 pixels, respectively, whereas the proposed method attains a substantially lower RMSE of 9.71 pixels. This corresponds to an error reduction of 5.50 pixels relative to DeepLabCut and 5.13 pixels compared to SLEAP. These results unequivocally demonstrate that the proposed semi-supervised temporal context-aware network delivers superior accuracy in fish pose estimation tasks, effectively mitigating prediction errors and enhancing model robustness.

**Figure 12:**
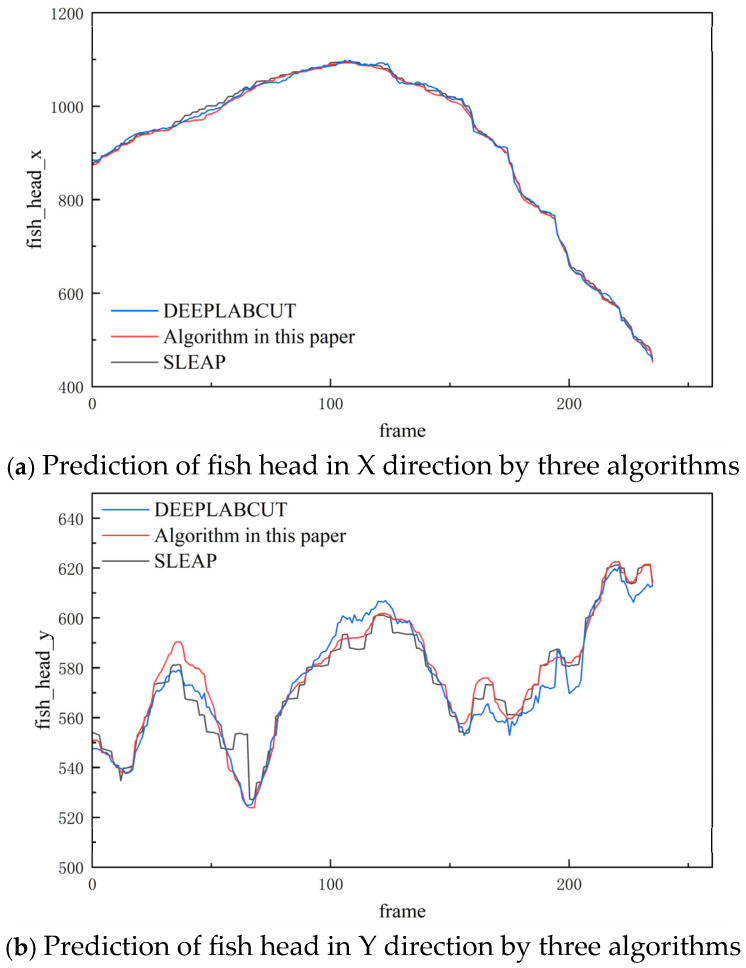
Prediction trajectories of key points of carp head by three networks.

Figure 12 illustrates the predicted trajectories of the head keypoint of a carp obtained from three different networks. The black curve represents predictions generated by the SLEAP network, the red curve corresponds to those from the DeepLabCut network, and the blue curve depicts the results of the proposed semi-supervised temporal context-aware algorithm. A comparative analysis of the figure reveals that the trajectory produced by the proposed method is markedly smoother and more continuous, exhibiting minimal jitter and absence of abrupt fluctuations relative to the predictions of SLEAP and DeepLabCut. This temporal stability in keypoint localization suggests enhanced robustness and higher reliability of the proposed algorithm in modeling fish pose dynamics over time. Such smoothness is critical in practical applications, as it reflects the model’s ability to maintain consistent tracking across frames, thereby reducing erroneous detections caused by occlusions, noise, or morphological ambiguities.

## 4. Discussion

Underwater biomimetic robotic fish have emerged as vital platforms for ocean exploration, offering unique advantages in environments that are otherwise inaccessible, hazardous, or costly for human divers and traditional equipment. Their bio-inspired locomotion provides higher maneuverability and energy efficiency compared to propeller-driven robots, making them particularly suitable for long-duration tasks such as environmental monitoring, biological observation, and seabed surveying. Within this broader context, high-precision fish pose estimation serves as a cornerstone technology. Accurate estimation of fish kinematics not only facilitates in-depth analysis of locomotion patterns and ecological adaptability but also provides critical guidance for the design optimization, real-time control, and autonomous navigation of robotic fish. Beyond robotics, precise pose estimation also holds promise for applications in intelligent fishery management, behavioral ecology, and marine biodiversity conservation.

This study tackles one of the most pressing challenges in aquatic pose estimation: the scarcity of large-scale, high-quality annotated datasets. The custom-designed fish motion visualization platform developed here represents a significant contribution toward filling this gap. By integrating dual synchronized cameras, auxiliary lighting, and a transparent tank setup, the platform allows high-resolution, multi-view recording of fish motion under controlled conditions. The resulting dataset not only provides fine-grained annotations of 21 biologically meaningful keypoints but also captures diverse swimming behaviors including straight, backward, and turning locomotion. This dataset constitutes a valuable benchmark resource for both supervised and semi-supervised pose estimation studies.

Building on this foundation, the proposed Semi-supervised Temporal Context-Aware Network (STC-Net) demonstrates how architectural innovations and loss design can jointly address the limitations of existing methods. The integration of a Bi-directional Convolutional Recurrent Neural Network (Bi-ConvRNN) introduces temporal continuity by incorporating contextual cues from preceding and succeeding frames. This design is particularly advantageous in aquatic environments, where self-occlusion, inter-fin interference, and motion blur frequently occur. In addition, the proposed unsupervised loss functions—temporal continuity loss and pose plausibility loss—enable effective utilization of unlabeled frames, thereby reducing dependence on manual annotation. This semi-supervised paradigm provides a scalable pathway for building robust models under real-world conditions where labeled data is inevitably limited.

Experimental results confirm that STC-Net achieves reliable performance under complex motion scenarios, maintaining robustness even when body parts are partially or fully occluded. Compared with fully supervised baselines, the proposed framework demonstrates superior generalization, highlighting the importance of incorporating temporal dynamics and biologically inspired constraints into pose estimation. Similar perspectives on the value of robust signal modeling have also been observed in other engineering domains. For example, Saleem et al. [31] proposed an AE-based pipeline monitoring approach combining Empirical Wavelet Transform with a customized one-dimensional DenseNet, illustrating the potential of advanced signal decomposition and deep learning for robust leak detection. Notably, similar perspectives on balancing model complexity and performance have been explored in recent studies on ballistic target recognition and aero-engine bearing fault diagnosis [32,33], which provide useful methodological references for our work.

Nevertheless, some limitations of this study warrant discussion. First, the dataset was collected in a controlled tank environment, which may not fully reflect the visual complexity of natural underwater habitats, where factors such as variable lighting, turbidity, and multi-species interactions occur. Future work should extend the framework to field deployments in open-water conditions to evaluate its robustness in natural ecosystems. Second, while the proposed STC-Net incorporates temporal context, the temporal window is currently limited. Exploring long-range temporal modeling, potentially via transformer-based architectures, may further enhance the ability to capture extended motion dependencies. Third, although carp were chosen as the representative species, fish morphology and swimming mechanics vary significantly across species. Cross-species validation and transfer learning approaches will be essential to ensure broader applicability. Finally, while the current focus is on single-fish pose estimation, real-world ecological and robotic applications often involve multiple interacting agents. Extending the framework to multi-fish pose tracking and interaction modeling remains an important direction.

In summary, this work contributes a novel dataset and a semi-supervised temporal context-aware network for fish pose estimation, providing a strong foundation for future advancements in underwater robotics and biological observation. By bridging biological insight, computer vision, and deep learning, this study highlights a promising pathway toward intelligent, adaptive, and scalable underwater robotic systems. Future research should emphasize ecological validity, cross-species adaptability, and real-world deployment, thereby advancing both fundamental biological understanding and the practical utility of biomimetic robotic fish.

## 5. Conclusions

This study focuses on the problem of fish pose estimation and proposes a novel algorithmic framework tailored to carp locomotion analysis. Initially, the limitations of conventional fully supervised animal pose estimation networks are examined, highlighting issues such as dependency on extensive labeled data and vulnerability to short-term occlusion or motion ambiguity. To address these challenges, two key enhancements are introduced. First, a semi-supervised learning strategy is employed by incorporating temporal continuity and pose plausibility loss functions. These unsupervised components enable the model to leverage unlabeled frames during training, enforcing biologically plausible and temporally consistent predictions. Deviations from these constraints result in penalization, which in turn guides the network toward learning more accurate and realistic spatiotemporal representations. Second, the network architecture is extended by embedding a temporal context module that incorporates frames immediately preceding and following the labeled frame. This design allows the model to capture short-term motion dynamics and resolve ambiguities caused by body part occlusions or similar appearance features, thereby improving estimation robustness in complex scenarios. Extensive ablation and comparative experiments conducted on the self-constructed dataset demonstrate that the proposed method significantly outperforms the standard fully supervised baseline and other state-of-the-art animal pose estimation approaches in terms of accuracy and generalization.

Our future research will focus on extending the proposed method to fish species with more complex and dynamic locomotion behaviors, such as swordfish or tuna. These species exhibit high-speed propulsion and sophisticated maneuvering capabilities, posing greater challenges for pose estimation but offering richer insights for the development of next-generation biomimetic robotic fish. In-depth analysis of their motion patterns could provide valuable design references for improving the agility, efficiency, and adaptability of underwater robotic systems operating in unstructured marine environments.

## Figures and Tables

**Table 1:** Performance of fully supervised networks.

NetWork	RMSE
ResNet_34	16.49
ResNet_50	14.50
ResNet_101	14.03

**Table 2:** Experimental platform and environment configuration.

Software Environment	PyCharm2020 + Anaconda3 + Python3.8 + Pytorch1.18.0	
hardware environment	Dell desktop computer	operating system: Windows 11
		processor: AMD Ryzen 7 5800H
		graphics card: NVIDIA GeForce RTX 3060Ti

**Table 3:** Ablation Experiment Results.

Network	Train Error (Pixel)	Val Error (Pixel)
Base	8.22	15.28
Semi-Supervised	7.95	15.36
Time-aware Contextual	6.49	14.67
Semi-Supervised Time-aware Contextual	6.98	9.71

**Table 4:** Comparison of different algorithms.

Algorithm	Train Error (Pixel)	Val Error (Pixel)
DeepLabCut [25]	12.33	15.21
SLEAP [26]	14.85	14.84
Algorithm in this paper	6.98	9.71

## Data Availability

The datasets used or analyzed during the current study are available from the corresponding author upon reasonable request.
